# Comparative Transcriptome Analysis of Toxic and Non-Toxic *Nassarius* Communities and Identification of Genes Involved in TTX-Adaptation

**DOI:** 10.3390/toxins12120761

**Published:** 2020-12-02

**Authors:** Shanmei Zou

**Affiliations:** College of Resources and Environmental Science, Nanjing Agricultural University, Nanjing 210095, China; zousm912@njau.edu.cn

**Keywords:** TTX, *Nassarius*, comparative transcriptome analysis, health, genes

## Abstract

*Nassarius* has caused serious people poisoning and death incident as a popular food due to tetrodotoxin (TTX) accumulation in their body. Understanding the genetic basis of tetrodotoxin (TTX) transformation and resistance in animals could lead to significant insights into adaptive evolution to toxins and toxin poisoning cures in medicine. Here we performed comparative transcriptome analysis for toxic and non-toxic communities in *Nassarius succinctus* and *Nassarius variciferus* to reveal their genetic expression and mutation patterns. For both species, the cellular and metabolic process, and binding and catalytic activity accounted for the top classification categories, and the toxic communities generally produced more up-regulated genes than non-toxic communities. Most unigenes and different expression genes were related to disease, e.g., heat shock protein and tissue factor pathway inhibitors, which involve detoxification and coagulation. In mutation levels, the sodium channel gene of *N. succinctus* had one amino acid mutation “L”, which is different from that of other animals. In conclusion, the comparative transcriptome analysis of different species and populations provided an important genetic basis for adaptive evolution to toxins, health and toxin poisoning cure research for TTX in marine gastropoda mollusk. Future studies will focus on the action mechanism of the important functional gene for TTX accumulation and resistance.

## 1. Introduction

*Nassarius*, a species-rich genus of Nassariinae, belonging to marine gastropod mollusks, is distributed in oceans globally, inhabiting inter- to subtidal shallow marine environments as facultative scavengers [1]. As scavengers, *Nassarius* are important in maintaining the balance of benthic community and are useful in the biomonitoring of Tributyltin (TBT) pollution in the marine environment [2]. *Nassarius* also has an imposex phenomenon that is considered to be the best biological indicator of TBT pollution in marine waters [3]. Except the environmental importance, for a long time, *Nassarius* is consumed as a popular food in Asian countries as an economic species [2], especially in China. However, in recent years, food safety problems have often existed in *Nassarius*. For example, eating *Nassarius* have caused hundreds of food poisoning and death incidents in China in the last several years, which has caused serious health issues [4,5]. Researchers have reported that the food poisoning incident was due to tetrodotoxin (TTX) accumulated in the edible tissues of individuals of the genus *Nassarius* [5]. Additionally, the toxicity varied in different *Nassarius* species and different populations with one species [2]. The origin of TTX accumulated in *Nassarius* is not determined yet, which mainly refers to whether *Nassarius* produces the TTX themselves or *Nassarius* obtains TTX from bacteria [6]. In either case, the accumulation mechanism and adaptive evolution to TTX is still unclear for *Nassarius.*

As a potent neurotoxin, TTX and Saxitoxin (STX) can specifically bind to sodium channels of animals by sharing one binding site with high affinities [7,8,9], which could lead to poisoning and death of animals. However, some animals can accumulate TTX, STX or other neurotoxins in their bodies to defend the external environment since they possibly could be resistant to toxins, such as in xanthid crab [10], blue-ringed octopus [11], and frogs [12,13,14,15]. Understanding how TTX is accumulated in animals and how animals resist TTX on the genetic level would be particularly critical for adaptive evolution research and toxin poisoning cures in medicine. Yotsu-Yamashita et al. ([16,17]) reported the distribution and localization of TTX-binding protein and their homologous proteins in puffer fish, which was possibly related to TTX transportation. Feroudj et al. ([18]) employed DNA microarray analysis to reveal some gene candidates related to TTX accumulation in puffer fish. However, the detailed accumulation mechanism of TTX in the animal body is unclear, and needs to be well understood in a wide range of animals. It has also been reported that the mutation in sodium channel genes can block the binding of toxins and can thus result in the toxin resistance, e.g., in snakes and frogs [12,16,19,20,21,22,23]. TTX often blocks the movement of sodium ions (Na^+^) across cell membranes and halts action potentials that control nerve impulses by binding to the outer pore of voltage-gated sodium channels in nerves and muscles [24]. In tetrodotoxic animals, the substitution of amino acids at the critical position of TTX-sensitive members of the sodium channel gene family or other positions in the outer pore could produce TTX resistance [25,26]. But we still do not know how marine gastropoda mollusk accumulate and resist TTX.

Currently, the genetic patterns among toxic and non-toxic *Nassarius* species and populations, involving TTX accumulation, transformation or resistance, is unknown. Here we performed comparative transcriptional analysis of both toxic and non-toxic communities in two *Nassarius* species (*Nassarius succinctus* and *Nassarius variciferus*), aiming to reveal their genetic expression patterns, identify candidate genes involving TTX accumulation and resistance, and finally provide a genetic basis for food and health safety in *Nassarius*.

## 2. Results

### 2.1. Quality of RNA-Seq Data and de Novo Transcriptome Assembly

A total of 12 samples were used for transcriptome sequencing in this study, half out of which were from *N. succinctus* and half of which were from *N. variciferus.* For each of *N. succinctus* and *N. variciferus*, 3 specimens (triple repeat) were toxic as a treat group and 3 specimens (triple repeat) were non-toxic as a control group. A total of 39.96 Gb bases and 40.19 Gb bases were generated for all specimens of *N. succinctus* and all specimens of *N. variciferus* respectively, based on Illumina Hiseq sequencing. The clean reads quality metrics after filtering sequences containing low-quality, adaptor-polluted and high content of unknown base (N) reads were shown in Table S1. The abundance of raw reads of the 12 specimens were from 55 to 63 Mb. The clean reads ratio of all specimens were from 70% to 80%. The total number of transcripts for two species was from 100,235 to 149,196, with a mean length from 430 bp to 576 bp and N50 length from 496 bp to 735 bp (Table 1). A total of 165,681 and 153,357 unigenes were obtained for *N. succinctus* and *N. variciferus*, respectively, with a mean length of 725 bp and 699 bp and an N50 length of 1160 and 1179 (Table 1). These reads and assembly quality indicated that the transcriptome sequencing performed well for functional analysis.

### 2.2. Gene Functional Annotation, CDS, SSR, and SNP Detection

**Figure 1:**
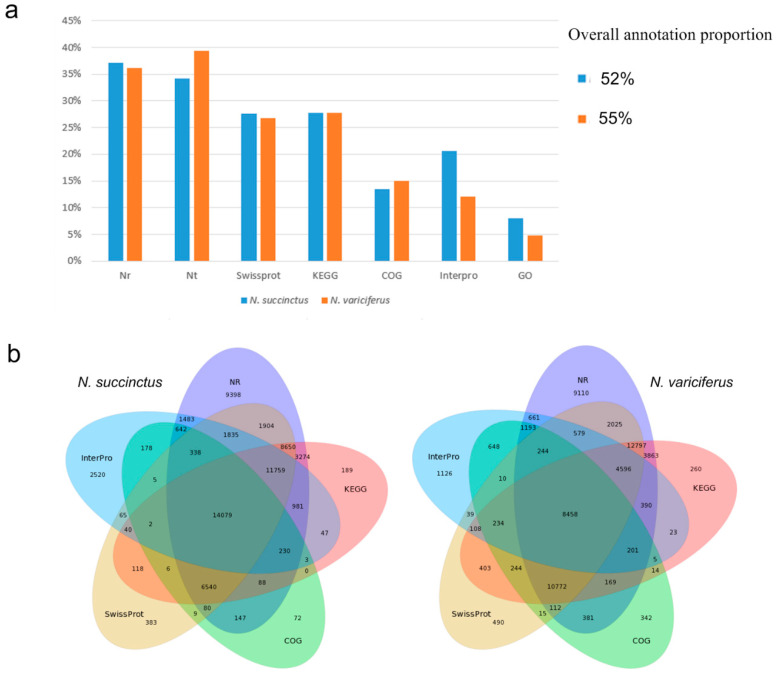
(**a**) Annotation proportion of unigenes to seven databases for two species; (**b**) Annotation difference of seven databases for *N. succinctus* and *N. variciferus*.

**Figure 2:**
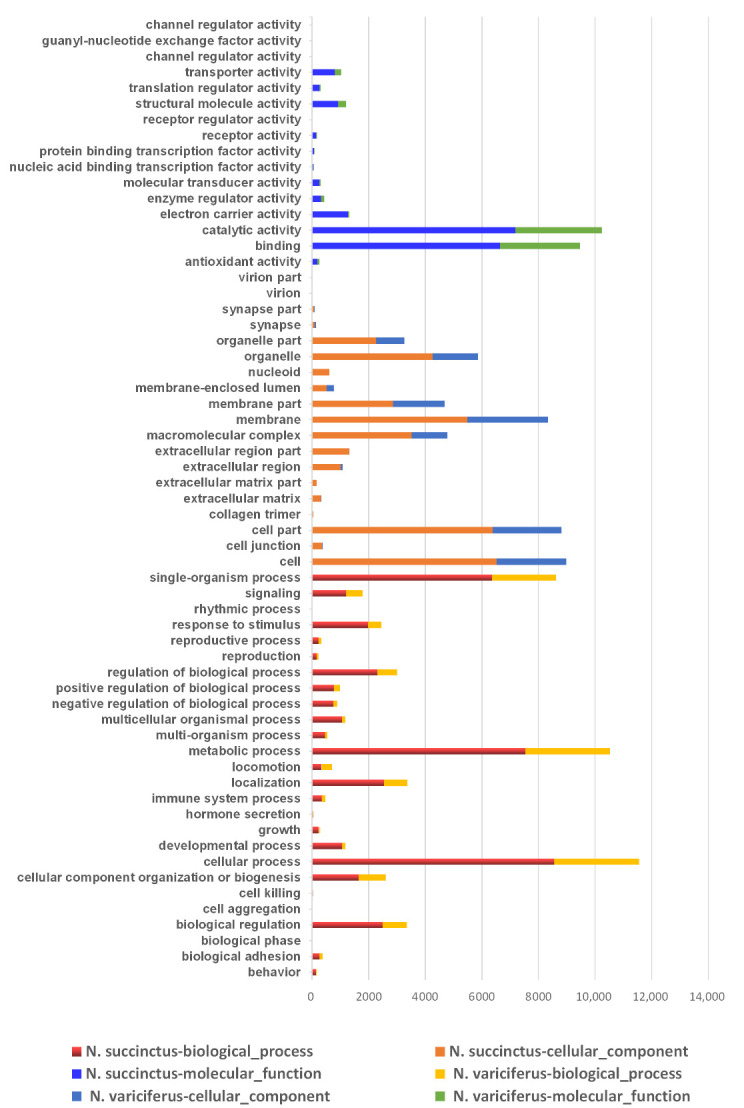
Distribution of gene ontology (GO) classification and functional enrichment for *N. succinctus* and *N. variciferus* respectively.

The unigenes of *N. succinctus* and *N. variciferus as* assembled were annotated to NR, NT, GO, COG, KEGG, Swissprot, and Interpro respectively. The unigene annotation for both *N. succinctus* and *N. variciferus* from the databases was generally consistent (Figure 1). For all annotated unigenes, the Nr, Nt, Swissprot, and KEGG databases got higher annotation proportions than the other databases (Figure 1a). The overall annotation proportion for *N. succinctus* and *N. variciferus* was 52% and 55% respectively, which meant that there were still some unigenes (48% and 45%) that could not be annotated to any databases. Among the 7 databases, the NR database got the most unique annotation for both species (Figure 1b). There were 14,079 and 8458 shared annotated genes among the 7 databases for *N. succinctus* and *N. variciferus*, respectively. The top annotated species were generally consistent between *N. succinctus* and *N. variciferus*, including *Aplysia californica*, *Oncorhynchus mykiss*, *Octopus bimaculoides*, *Monosiga brevicollis MX1*, *Lottia gigantea*, *Biomphalaria glabrata*, *Crassostrea gigas*, *Trichuris suis*, *Exaiptasia pallida*, *Lingula anatine*, and *Mus musculus*, where *A. californica* showed the highest annotation proportion (Figure S1). The Gene Ontology (GO) classification and functional enrichment was performed for unigenes, including three ontologies of molecular function, cellular components and biological processes. *N. variciferus* and *N. succinctus* produced coincident patterns of functional enrichment, for which the cellular and metabolic process from the biological process, and binding and catalytic activity from the molecular function accounted for the top classification categories (Figure 2).

The coding sequence (CDS) was selected from the segment of unigenes that best mapped to the functional databases. For both *N. succinctus* and *N. variciferus*, the mean length of CDS was 300–500 bp and the fragments with 300 bp made up the highest proportion (Figure S2). Among all the repeat nucleotide types of SSR detection, the Di-nucleotide type made up the highest proportion, followed by AC/GT and Tri-nucleotide for both species (Figure S3). Both species produced similar SNP types, where the transition was more than transversion and the toxic samples generally produced slightly more SNP than non-toxic samples (Figure S4).

### 2.3. Genetic Expression Patterns, Different Expressed Genes, and qRT-PCR Validation

**Figure 3:**
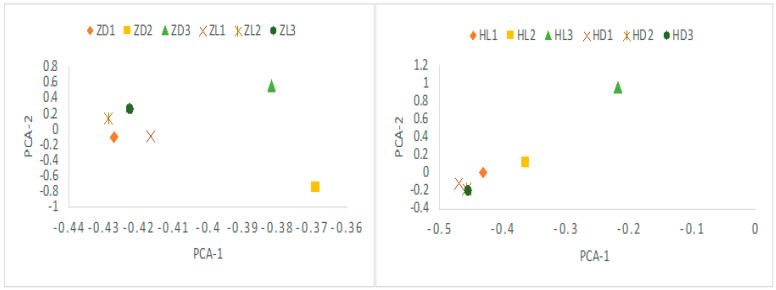
PCA plot for the difference of expression levels between the toxic and non-toxic communities (ZD and HD: non-toxic specimens, ZL and HL: toxic specimens). It indicated that the toxic and non-toxic communities generally show similar expression patterns. ZD: *N. variciferus* from Dalian. HD: *N. succinctus* from Dalian. ZL: *N. variciferus* from Lianyungang. HL: *N. succinctus* from Lianyungang.

**Figure 4:**
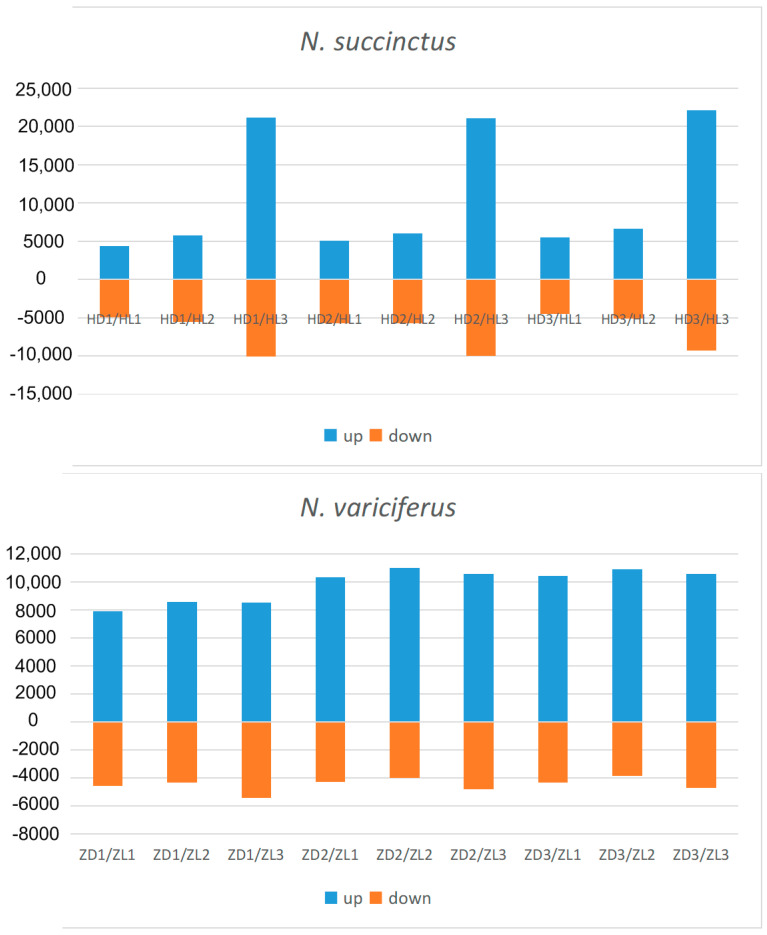
Statistics of up-regulated and down-regulated genes between toxic and non-toxic specimens of *N. succinctus* and *N. variciferus.* HD and ZD represent non-toxic samples in *N. succinctus* and *N. variciferus* respectively from Dalian, which were set as a control group. HL and ZL represent toxic samples in *N. succinctus* and *N. variciferus* respectively from Lianyungang. HDn, HLn, HLn, and ZLn represent the order number of samples in *N. succinctus* and *N. variciferus.* HL and ZL were set as treatment group. Examples: HD1-VS-HL3 means that the HL3 generated more up-regulated genes (blue) than down-regulated (orange) in comparison with HD1. ZD1-VS-ZL3 means that the ZL3 generated more up-regulated genes (orange) than down-regulated (blue) genes in comparison with ZD1.

**Figure 5:**
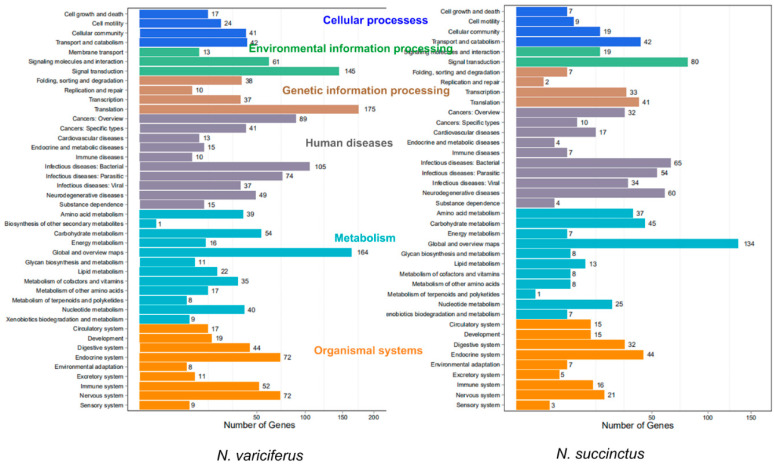
KEGG annotation for the unigenes of two species.

After assembly and mapping clean reads to unigenes, the gene expression levels for each sample was first calculated by PCA analysis. As shown in Figure 3, the expression level between the toxic and non-toxic specimens was generally different for both species. Two toxic *N. succinctus* specimens from Lianyungang showed more similar gene expression patterns. Two non-toxic specimens of *N. variciferus* from Dalian showed different expressions from the non-toxic samples in Lianyungang. The differently expressed genes (DEGs) revealed up-regulated and down-regulated genes between toxic and non-toxic specimens for both species (Figure 4). For *N. variciferus*, compared with the non-toxic samples (ZD1-ZD3), the toxic samples (H1-H3) generally produced more up-regulated genes. For *N. succinctus*, two toxic specimens (HL2 and HL3) also produced more up-regulated genes in comparison with the non-toxic specimens (HD1 and HD3). For both species, the KEGG annotated the unigenes into six categories, including cellular processes, environmental information processing, genetic information processing, human disease, metabolism, and organism systems (Figure 5), where the signal transduction, global and overview maps, translation and infectious disease accounted for the top annotation. Thereinto, the human disease category included the neurodegenerative disease annotation.

**Figure 6:**
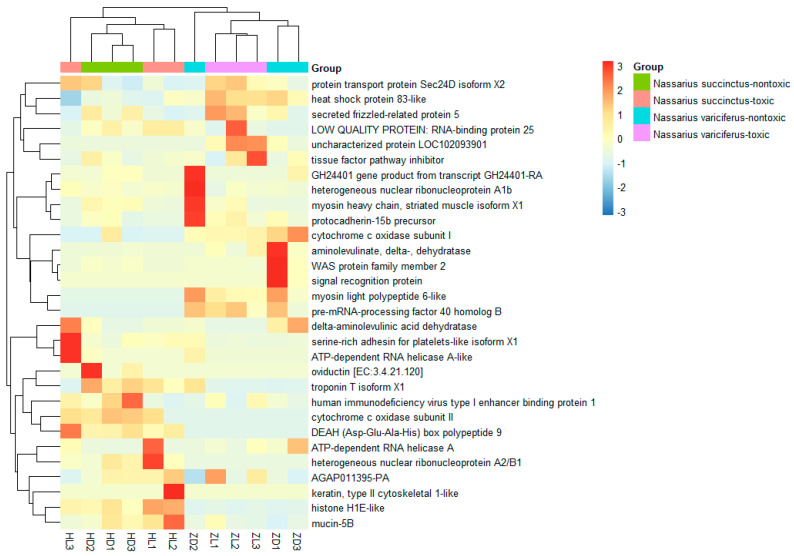
Expression comparison of DEGs between the toxic and non-toxic communities of two species.

**Figure 7:**
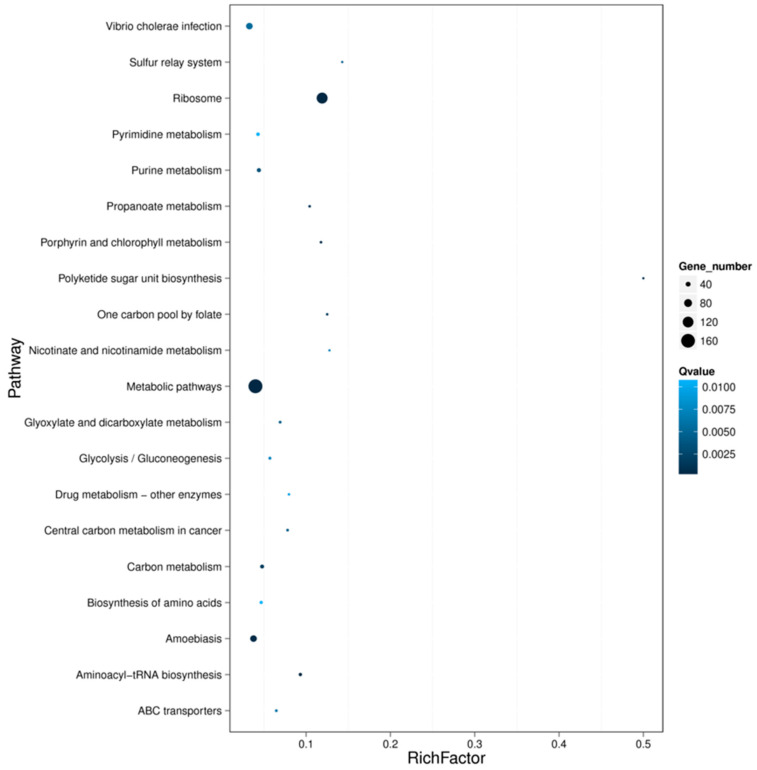
The pathway functional enrichment of the DEGs for *N. succinctus.*

The most common differently expressed genes were obtained from toxic (treatment) and non-toxic (control) groups for both species (Figure 6). It was shown that the most different DEGs were divided into two clades clearly for the two species. Compared with the non-toxic groups, the most upregulated genes from toxic groups included heat shock protein 83-like, coagulation, RNA-binding protein, tissue factor pathway inhibitor, ATP-dependent RNA helicase, serine-rich adhesion for platelets-like insoform, heterogeneous nuclear ribonucleoprotein, cytochrome c oxidase subunit II and others. The pathways related to the main upregulated genes were protein processing in endoplasmic reticulum, spliceosome, complement and coagulation cascade, Alzheimer’s disease, Parkinson’s disease, Huntington’s disease and some unknown pathways. On the other hand, there were also some genes that were upregulated in the non-toxic samples (Figure 6). The pathway functional enrichment results indicated that most DEGs referred to metabolic pathways for both species, together with some disease pathways like Alzheimer’s disease, Amoebiasis, Huntington’s disease and drug metabolism (Figure 7, Figure S5).

**Figure 8:**
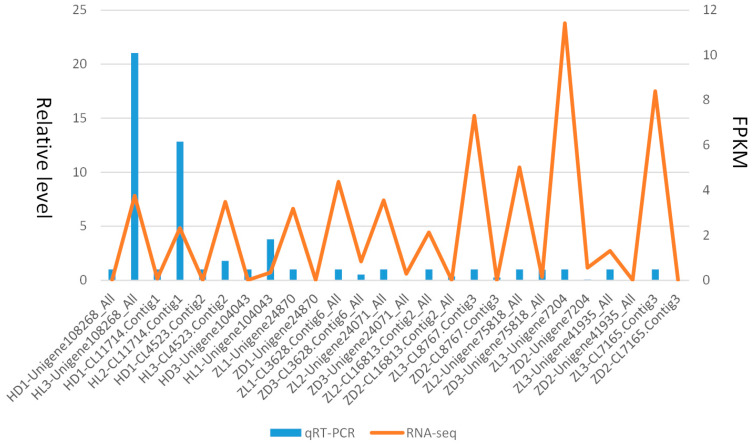
Consistency verification of relative expression levels from RT-PCR and actual expression levels from RNA-seq.

Multiple DEGs mostly differently expressed in two species were selected randomly for qPCR, where the non-toxic sample was set as a control compared with the toxic sample for each gene. The 18S was selected as the reference gene. In general, the statistic results indicated that the relative expression level of the unigenes by qPCR was generally consistent with the actual expression level (FPKM) for treat and control groups (Figure 8). The detailed primers for qPCR of the DEGs were listed in Table 2.

### 2.4. Mutation in Sodium Channel Genes

**Figure 9:**
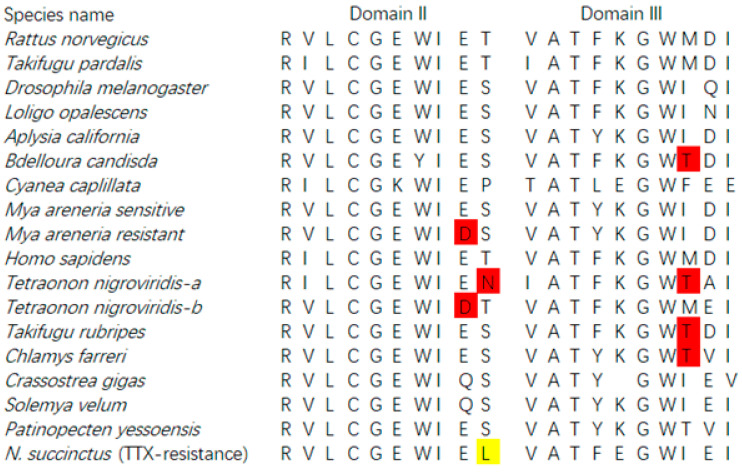
Potential amino acid mutation site related with TTX resistance on sodium channel of *N. succinctus.* The site indicated in yellow is newly identified by our study.

Based on the transcript unigenes of both species, we tried to retrieve the sodium channel genes for both species. A total of 3425 bp sequences (unigene CL8899.Contig1_All) from *N. succinctus* was best matched to the sodium channel genes in the reference database with an identified score of more than 80%. The amino acids of this matched unigene was identified as Domain II and Domain III of sodium channels. Among the amino acid sites, we found one new amino acid “L” in Domain II, in comparison with all other species (Figure 9). By PCR confirmation, we obtained the same sequence of this newfound sodium channel gene from *N. succinctus*. Unfortunately, no perfect matched sodium channels genes were found from the unigenes of *N. variciferus* even though we blasted the clean reads of both species (150 bp) to all reference sequences, which meant that the sequences of Domain I and Domain IV could not be obtained in this study (the reason for this was referred to in the discussion). The Genbank accession number of sodium channel genes obtained in this study was MT989337.

## 3. Discussion

More and more research has pointed out that animals can accumulate toxins, like PSP (paralytic shellfish poisoning), Saxitoxin (STX) and TTX in their body and thus produce toxin resistance [27,28]. It is significant to reveal the genetic basis of toxin accumulation and resistance in various animals accumulating toxins, not only for the understanding of adaptive evolution, but also for food-poisoning cures in medicine. Populations occupying different environments within one species could have a different toxicity with neurotoxins, like frogs (with epibatidine) [12] and *Nassarius* (with TTX). Our previous studies showed that different populations in *N. succinctus* and *N. variciferus* have different toxicity with TTX [29]. So in this study, both toxic and non-toxic communities from *N. succinctus* and *N. variciferus* were used for transcriptome analysis to reveal their genetic expression patterns and mutations. Two species were compared for obtaining a comprehensive genetic basis.

First of all, *N. succinctus* and *N. variciferus* showed similar genetic annotation, where just half of the unigenes were annotated to the seven databases. This is not strange since no genome or transcriptome analyses are performed for *Nassarius* yet. But both species demonstrated similar genetic expression patterns. For both species, the cellular and metabolic process from the biological process, and binding and catalytic activity from molecular function accounts for the top classification categories. As a potent neurotoxin, TTX mainly causes poisoning in animals by binding the sites of sodium channels [9], which suggests that the metabolic process, binding and catalytic activity possibly play a key role in the transformation of TTX. For both species, the toxic communities generally produced more up-regulated genes than the non-toxic communities. The PCA analysis of DEGs also indicated that the gene expression patterns of toxic specimens was slightly different from that of non-toxic specimens for both species. These may suggest that special metabolic activities associated with TTX accumulation and resistance occur in toxic samples. Particularly, the samples in Lianyungang showed more different expression genes from that in Dalian. Maybe this is related to the different marine environments between Dalian and Lianyungang, which have distinct bays. Because of the origin of TTX in *Nassarius*, this is possibly from the food web which could be diverse among different marine environments. This also could explain that the toxicity varies within different populations of some *Nassarius.* The KEGG annotation showed that most DEGs were related with environmental information processing, human disease and metabolism. Particularly, the neurodegenerative disease annotation was included in the human disease category. This is consistent with the poisoning symptom in humans by TTX.

The pathway functional enrichment also verified that some DEGs referred to disease pathways like Alzheimer’s disease, Amoebiasis, Huntington’s disease and Drug metabolism. Furthermore, we selected the most common DEGs between the toxic and non-toxic groups for both species. It was indicated that the pathways of the most differently expressed genes were also related to detoxification and human disease, like heat shock protein and cytochrome c oxidase subunit. The heat shock protein involves the pathway of protein processing in the endoplasmic reticulum. One of the most important functions of endoplasmic reticulum is detoxification, which is the removal of all the toxic materials such as metabolic waste or drugs [30,31]. This strongly suggests that the TTX present in the tissues of *Nassarius* is also possibly removed by a special mechanism. Cytochrome c oxidase subunit I and II involve the pathways of various diseases like oxidative phosphorylation, Metabolic pathways, cardiac muscle contraction, non-alcoholic fatty liver disease (NAFLD), Alzheimer’s disease, Parkinson’s disease, and Huntington’s disease. The cytochrome c oxidase subunit plays a key role in respiration. It is possible that the cytochrome c oxidase subunit is also related to the metabolic mechanism of TTX.

Finally, all the transcriptome unigenes were clustered against the sodium channel gene references to get the sodium channel genes of *Nassarius.* We obtained D2 and D3 domains of *N. succinctus* with a length of 3425 bp where one amino acid site “L” was different from that of all the other animals possessing TTX resistance. Whether this new amino acid “L” is a potential mutational site with TTX resistance should be further verified by electrophysiology as some studies did in frogs [12]. Unfortunately, other domains of *Nassarius* could not be obtained by unigene blasting, reads blasting, or RACE-PCR. We infer that the reasons for this may be that the RNA-seq sequences in this study do not completely cover the sodium channel regions of both species due to the complication of the sodium channel family. In future studies, we would like to employ comprehensive samples and deep sequencing to explore the complete sodium channel genes of various toxic and non-toxic communities in more *Nassarius* species to better understand the adaptive evolution of TTX in *Nassarius*.

### Availability of Data and Materials

The Illumina RNA-seq data generated from *N. succinctus* and *N. variciferus* are available from the NCBI SRA database (http://trace.ncbi.nlm.nih.gov/Traces/sra) under accession SRR10582953-SRR10582964. The Genbank accession number of sodium channel genes obtained in this study is MT989337.

## 4. Materials and Methods

### 4.1. Sample Collection, Treatment and Toxicity Test

**Figure 10:**
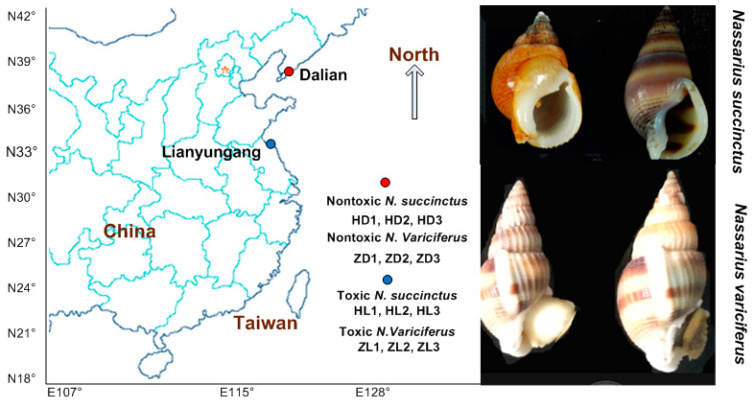
Toxic and non-toxic communities of *N. succinctus* and *N. variciferus* collected from the China coast, which were collected together with samples from our previous study (Zou et al. 2019), which included detailed sample collection and TTX toxicity detection.

The TTX toxicity of both *N. succinctus* and *N. variciferus* was tested in our previous research, which included a detailed sample collection and toxicity detection [29]. Based on the toxicity of all samples of various *Nassarius* species tested in [29], we selected samples of *N. succinctus* and *N. variciferus* for transcriptional analysis since they included both toxic and non-toxic communities from Dalian and Lianyungang. The samples used in this study were collected together with that in [29]. Since the RNA-seq requested a high quality of RNA, we had to use fresh samples without TTX detection again. The liver and pancreas were treated for RNA extraction and sequencing since they showed a higher toxicity in animals accumulating TTX [15,32,33]. All the samples in this study were stored by liquid nitrogen for the next RNA extraction. For the RNA-seq protocol, the toxic and non-toxic specimens were sequenced as treatment groups and control groups respectively for each species. We selected three toxic specimens and three non-toxic specimens as triple repeats for the treatment group (toxic group) and the control group (non-toxic group) respectively, for each of *N. succinctus* and *N. variciferus*. The detailed sample collection is shown in Figure 10.

### 4.2. RNA Isolation, Library Construction, and RNA-Seq

Total RNA was extracted separately from the tissues of each sample by the CTAB method [34]. The yield and quality of RNA was determined by agarose gel electrophoresis (AGE) and a Nanodrop 2000 instrument. Next, the mRNA was fragmented with a fragmentation buffer and was used as a template for synthesizing the first cDNA chain with random hexamers as a six-base random primer. Then double-stranded cDNAs were performed using these short fragments as templates by RNase H and DNA polymerase I. The AMPure XP beads were used for purifying the double-stranded cDNA, and after, end repair adapters were ligated to these short fragments using T4 DNA ligase (Invitrogen, Carlsbad, CA, USA). Finally, products were enriched by PCR to generate the cDNA library. After the completion of the library construction, Qubit 2.0 and Agilent 2100 was used to detect the library’s concentration and the size of the inserted fragments. The effective concentration of the library was accurately quantified with Q-PCR. Finally, after being qualified, the library was sequenced using Illumina HiSeq 4000 [35].

### 4.3. Data Preprocessing and Transcriptome Assembly

Before downstream analyses we filtered reads that contained low-quality, adaptor-polluted and high content of unknown base (N) reads using internal software, following the criterion: (1) remove reads with adaptors; (2) remove reads where unknown bases (N) are more than 5%; (3) remove the reads in which the percentage of base quality is less than 15 times greater than 20%. After filtering, the remaining reads were stored in a FASTQ format for downstream analysis. The Trinity (v2.0.6) [36] was used to perform de novo assembly. The assembly quality was evaluated with N50 and ortholog hit ratios (OHR). Paired reads passing the filter were then concatenated using Concatenate datasets (version 1.0.0) in both the right and left direction. Transcript abundancies were calculated by RSEM version 1.1.1754 [37,38] using the pool of non-normalized reads with default settings.

### 4.4. Gene Annotation and Expression, DEG, CDS, SSR, and SNP Detection

The functional annotation was performed from databases of NT, NR, GO, COG, KEGG, SwissProt and InterPro. With BLASTX, the final assembly was submitted to these databases with the threshold of E-value ≤ 10−5 [39]. The NR and InterProScan5 were used to get GO annotation and InterPro annotation respectively. Unigenes were obtained from mapping clean reads to unigene datasets by Bowtie228 (version 2.1.0, http://bowtie-bio.-sourceforge.net/bowtie2/index.shtml) at a sensitive setting. The gene expression level (FPKM values for each unigene) was calculated with RSEM (version 1.2.29) [38] with default parameters. We detected DEGs (Differentially Expressed Genes) with Possion Dis as requested [40] where genes with|log2ratio| ≥ 1 and false discovery rate (FDR) < 0.05 were identified as DEGs. The PCA analysis was performed with all samples by princomp, a function of R. With functional annotation, we selected the segment of unigene that best mapped to functional databases in a priority order of NR, SwissProt, KEGG, COG as its CDS, which was displayed from 5′ to 3′ in a FASTA format. For some unigenes that could not be aligned to any database mentioned above, the ESTScan was used to predict the CDS [41]. The MISA [42] was used to find SSR in unigenes and the Primers5 was used to design primers for each SSR. Finally, the GATK was used to call SNP variants for each sample with unigenes as a reference, and the results were stored in a VCF format [43].

### 4.5. Validation of DEGs by qRT-PCR

The quantitative real-time PCR (qRT-PCR) was performed to further validate the confidence of the high-throughput transcriptome sequencing. A total of thirty random differentially expressed genes were selected for qPCR using the SG Fast qPCR Master Mix (High Rox) using the cDNA synthetized above, where the toxic (treat) and non-toxic (control) samples were compared for each gene. The Primer Premier 5.0 was used for designing primers for the genes selected. PCR reactions were carried out in a total volume of 20 μL, including 10 μL of SybrGreen qPCR Master Mix. PCR conditions for all primer sets were as follows: 95 °C for 3 min, 45 cycles of 95 °C for 7 s, 57 °C for 10 s, 72 °C for 15 s, which was followed by disassociation curve analysis in ABI Stepone plus (Applied Biosystems, Foster City, CA, USA). The 18S gene was used as an internal reference. The 2-△△Ct method was used to calculate the relative gene expressions of the samples, which were normalized using the 18S mRNA level. The SPSS was used to determine whether the expression data held any significant differences at the *p* < 0.05 level. The fold changes of these genes in toxic and non-toxic communities were calculated via FPKM. The genes’ log2 fold change values of qRT-PCR and RNA-Seq were used for graphical presentation.

### 4.6. Detection of Sodium Channels Genes

Based on the transcript unigenes of all samples, we retrieved the sodium channel genes to which the TTX binds. Most available **s**odium channels genes of mollusk, humans, mice, and others from NCBI were downloaded as a reference database. Then all unigenes of *N. succinctus* and *N. variciferus* were mapped to the reference database by blasting. The unigene that was perfected matched to the reference sequences with the highest similarity score was considered as a candidate for the sodium channel genes of *Nassarius*. Next, we designed primers from the candidate sodium channel genes of *Nassarius* for PCR validation. Finally, the correct sodium channel genes were submitted to the Genbank.

## Figures and Tables

**Table 1:** Quality statistics of transcripts and unigenes for *N. succinctus* (H) and *N. variciferus* (Z).

Sample	Total Number	Total Length (bp)	Mean Length (bp)	N50	GC (%)
**Transcripts**					
HL1	102,960	53,331,724	517	616	41.81
HL2	105,347	55,526,297	527	632	41.67
HL3	139,998	80,687,307	576	735	43.02
HD1	100,186	54,213,432	541	664	41.44
HD2	109,003	58,814,502	539	664	42.32
HD3	100,235	52,686,293	525	633	41.76
ZL1	127,934	59,028,848	461	564	40.73
ZL2	146,655	67,758,333	462	568	40.74
ZL3	149,196	66,729,017	447	534	40.7
ZD1	120,572	54,632,419	453	538	40.82
ZD2	107,543	47,553,693	442	521	41.1
ZD3	127,451	54,918,390	430	496	41.11
**Unigenes**					
HL1	67,940	39,732,836	584	773	42.26
HL2	68,665	41,007,566	597	795	42.16
HL3	88,796	60,545,304	681	1001	43.49
HD1	66,739	41,080,421	615	844	41.76
HD2	72,368	44,546,054	615	841	42.79
HD3	69,148	40,744,957	589	783	42.09
H-All-Unigene	165,681	120,168,668	725	1160	43.13
ZL1	65,474	40,591,850	619	879	41.04
ZL2	74,256	46,318,502	623	894	41.06
ZL3	75,204	45,575,216	606	854	40.99
ZD1	66,720	38,813,305	581	790	41.01
ZD2	59,005	33,677,252	570	768	41.28
ZD3	68,165	38,195,019	560	740	41.37
Z-All-Unigene	153,357	107,209,071	699	1179	40.9

**Table 2:** Primers for quantitative real-time PCR, gene annotations, and fragments amplified.

Gene ID	Length (bp)	Primer Sequence (5′-3′)	Annotation
Unigene108268_All			
CL11714.Contig1	127	F: AGGGCTTCTGAGAGGGTGG	testicular haploid expression protein
		R: GCAGTCTAAGGGAGGCAACAT	
CL4523.Contig2	111	F: CCATACCTTACAGCCAACTCATT	basic proline-rich protein-like
		R: GCTTCGTGCCTTCGTTCTT	
Unigene104043	135	F: GCTGCGGAAGGTGTCTATGT	Cytochrome P450
		R: CTCAACTTTGTGCCGATGC	
Unigene24870	224	F: ATGCCCATTGTGGACCCTA	NA
		R: CCTAATGGAAGACCACCACCTA	
CL3628.Contig6_All	188	F: TTTGGCTCATTCGCCTGTA	coagulation factor II
		R: GTCTATTCCGCTTCTTCTCACTC	
Unigene24071_All	149	F: GTACAAGTAATAGTGTCATTGT	myosin binding subunit
		R: TCGGATGATAAGAATTT	
CL16813.Contig2_All		F: GTCATGTGACTTGTCATGTG	carnitine O-palmitoyltransferase 1
		R: CTACGCAACAGGTTGTAATATG	
CL8767.Contig3	170	F: CTTCTGCACAGACCGACCAT	NA
		R: ACAACAGCCAGCCAACACTAT	
Unigene75818_All	189	F: TGCCTACTAGAGCCTGCTA	RNA-binding protein 25-like
		R: CCTACCTCGAAAGTGGTGTT	
Unigene7204	164	F: TAGCCCAGCCGACTATGAAA	coagulation factor V
		R: CTCACACAACACGCCACACT	
Unigene41935_All	158	F: ATGGGCTGCTGTTGTTTCGTA	NA
		R: GCAGATGGACATGACGCCAC	
CL7165.Contig3	135	F: CCACCTTGGCGTTGATGTT	antigen-like
		R: CGTCCACCTACGGCTATCTT	
18S	126	F: CATCTTTCAAATGTCTGCCCTA	
		R: TGGATGTGGTAGCCGTTTCT	

## Supplementary Materials

The following are available online at https://www.mdpi.com/2072-6651/12/12/761/s1, Table S1 Statistics for reads filter in *N. succinctus* and *N. variciferus*. Figure S1: Distribution of annotated species for both *N. succinctus* and *N. variciferus* which show similar annotated patterns. The Aplysia California species take accounted for the top proportion. Figure S2: Length distribution of CDS from unigenes of *N. succinctus* and *N. variciferus*, respectively. Figure S3: SSR nucleotide types for *N. succinctus* and *N. variciferus*, respectively. Figure S4: SNP variation types for *N. succinctus* and *N. variciferus*, respectively. Figure S5: The pathway functional enrichment of the DEGs for *N. variciferus*.
