# Supplementary material for: Comparative Transcriptome Analysis of Toxic and Non-Toxic Nassarius Communities and Identification of Genes Involved in TTX-Adaptation
Source: Toxins (Basel). 2020 Dec 2;12(12):761. doi: 10.3390/toxins12120761 (PMC7761612; doi:10.3390/toxins12120761)
Supplement: Supplementary file 1 [file toxins-12-00761-s001.pdf]

toxins

Supplementary Materials: Comparative Transcriptome Analysis of Toxic and Non-Toxic *Nassarius* Communities and Identification of Genes Involved in TTX-Adaptation

Shanmei Zou

Table S1. Statistics for reads filter in *N. succinctus* and *N. variciferus.*

Sample	Total Raw Reads(Mb)	Total Clean Reads(Mb)	Total Clean Bases(Gb)	Clean Reads Q20(%)	Clean Reads Q30(%)	Clean Reads Ratio(%)
N. succinctus						
HL1	58.37	44.66	6.7	98.09	94.47	76.52
HL2	59.9	44.32	6.65	97.88	93.98	73.99
HL3	55	44.12	6.62	98.27	94.7	80.22
HD1	60.43	44.31	6.65	98.19	94.59	73.33
HD2	60.55	44.24	6.64	98.42	95.42	73.06
HD3	62.14	44.67	6.7	98.45	95.53	71.89
N. variciferus						
ZL1	63.16	44.59	6.69	96.85	90.95	70.6
ZL2	63.16	44.47	6.67	96.75	90.71	70.42
ZL3	63.16	44.86	6.73	96.74	90.65	71.04
ZD1	59.92	44.65	6.7	96.88	91.04	74.53
ZD2	59.92	44.91	6.74	96.86	91.05	74.95
ZD3	61.54	44.41	6.66	96.85	91.03	72.16

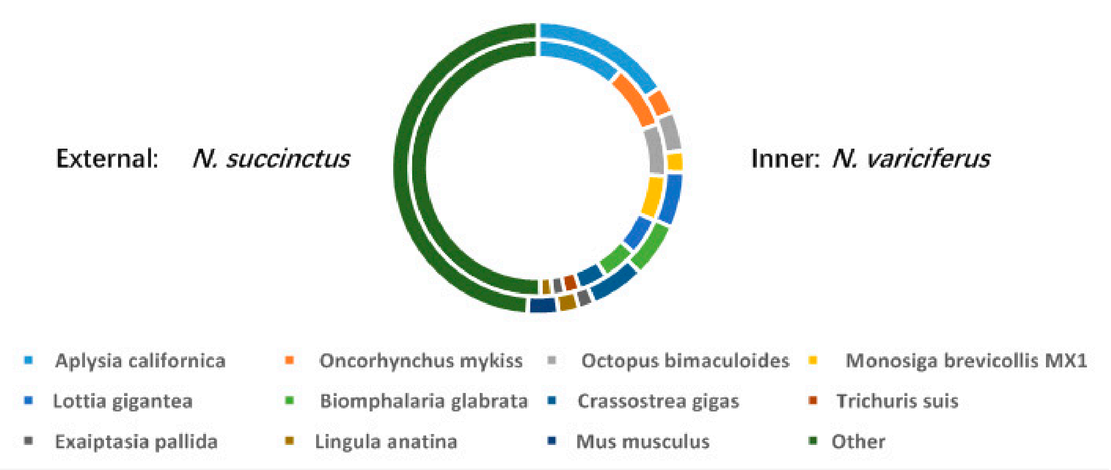

Figure S1. Distribution of annotated species for both *N. succinctus* and *N. variciferus* which show similar annotated patterns. The Aplysia california species take accounted for the top proportion.

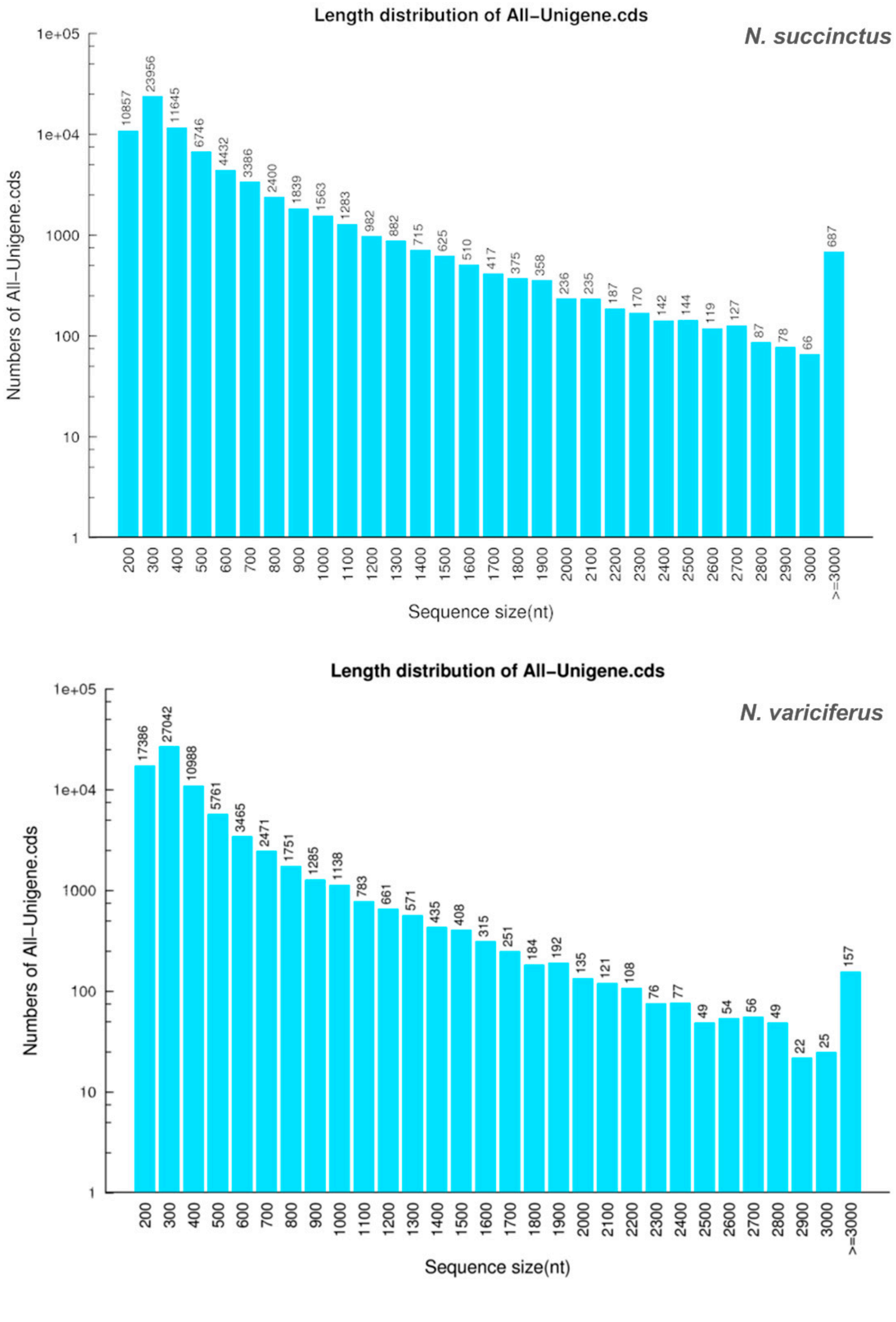

Figure S2. Length distribution of CDS from unigenes of *N. succinctus* and *N. variciferus* respectively.

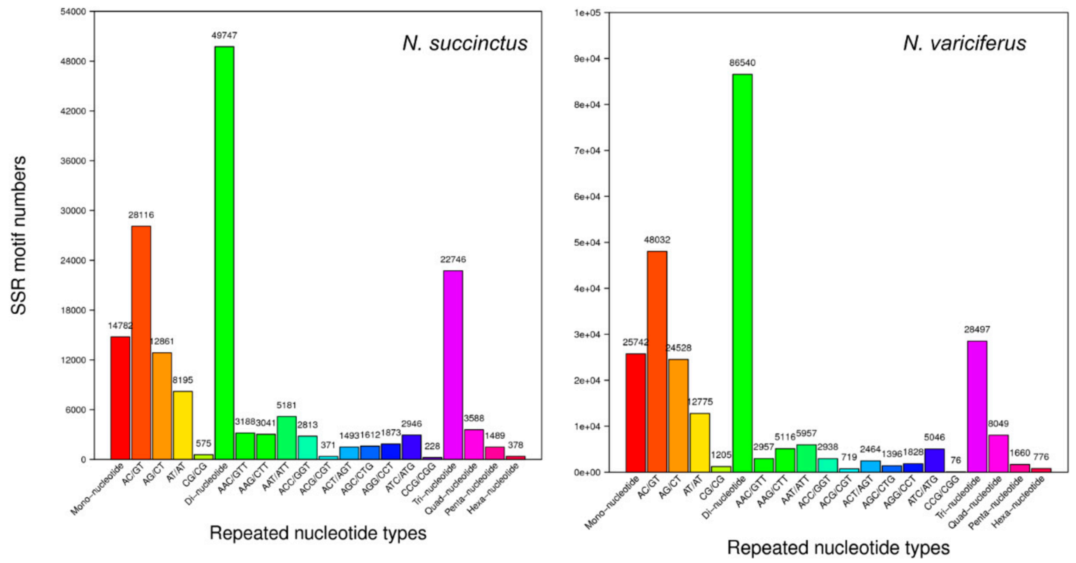

Figure S3. SSR nucleotide types for *N. succinctus* and *N. variciferus* respectively.

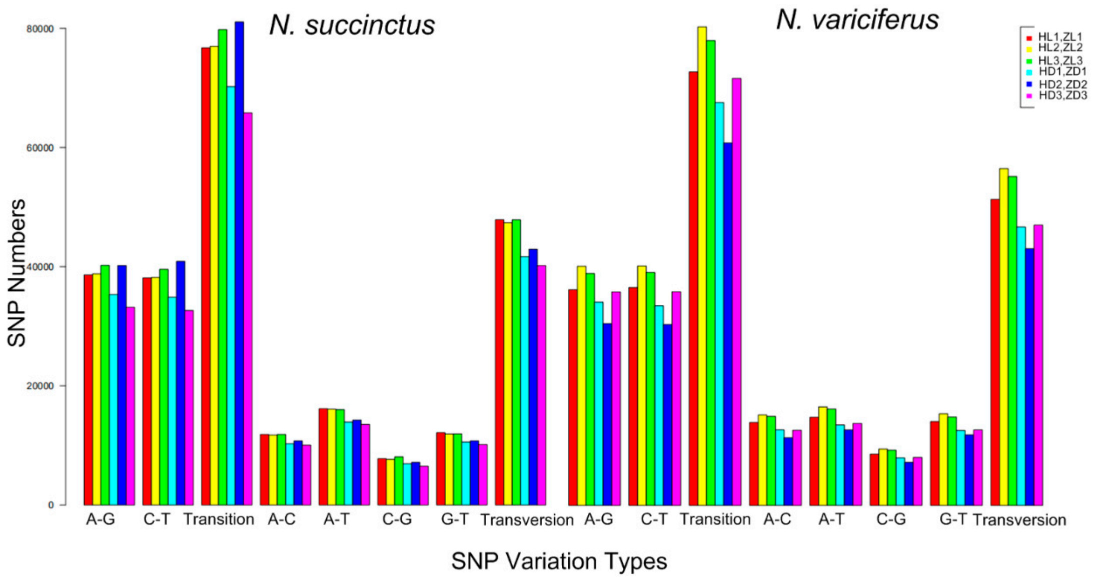

Figure S4. SNP variation types for *N. succinctus* and *N. variciferus* respectively.

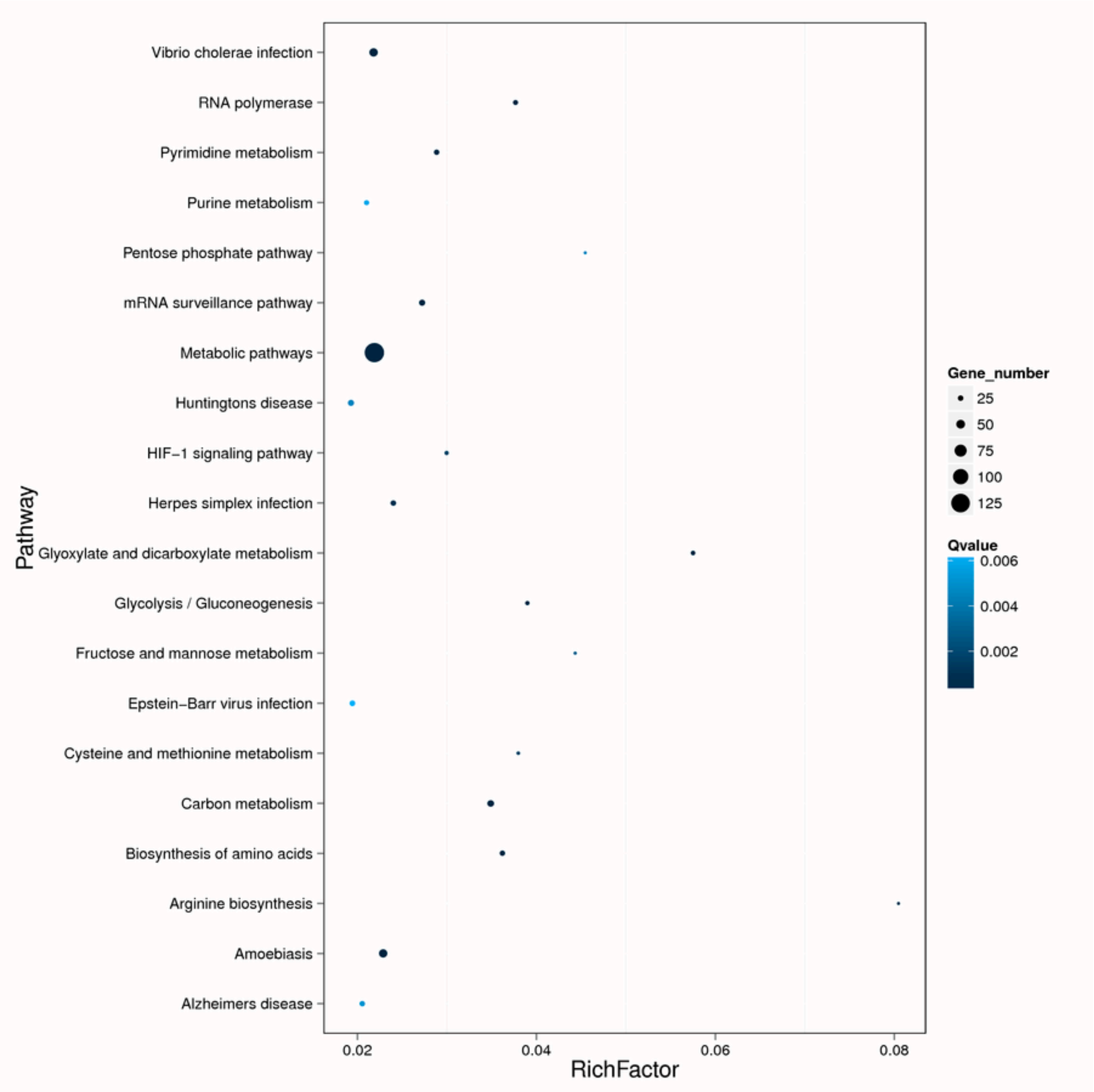

Figure S5. The pathway functional enrichment of the DEGs for *N. variciferus*.
